# The atrial secondary tricuspid regurgitation is associated to more favorable outcome than the ventricular phenotype

**DOI:** 10.3389/fcvm.2022.1022755

**Published:** 2022-11-29

**Authors:** Mara Gavazzoni, Francesca Heilbron, Luigi P. Badano, Noela Radu, Andrea Cascella, Michele Tomaselli, Francesco Perelli, Sergio Caravita, Claudia Baratto, Gianfranco Parati, Denisa Muraru

**Affiliations:** ^1^Department of Cardiology, Istituto Auxologico Italiano, IRCCS, Milan, Italy; ^2^Department of Medicine and Surgery, University of Milano-Bicocca, Milan, Italy; ^3^Emergency University Hospital Bucharest, University of Medicine and Pharmacy Carol Davila Bucharest, Bucharest, Romania; ^4^Department of Management, Information, and Production Engineering, University of Bergamo, Dalmine, Italy

**Keywords:** atrial secondary tricuspid regurgitation, atrial fibrillation, heart failure hospitalizations, secondary tricuspid regurgitation, prognosis

## Abstract

**Aim:**

We sought to evaluate the differences in prognosis between the atrial (A-STR) and the ventricular (V-STR) phenotypes of secondary tricuspid regurgitation.

**Materials and methods:**

Consecutive patients with moderate or severe STR referred for echocardiography were enrolled. A-STR and V-STR were defined according to the last ACC/AHA guidelines criteria. The primary endpoint was the composite of all-cause death and heart failure (HF) hospitalizations.

**Results:**

A total of 211 patients were enrolled. The prevalence of A-STR in our cohort was 26%. Patients with A- STR were significantly older and with lower NYHA functional class than V-STR patients. The prevalence of severe STR was similar (28% in A-STR vs. 37% in V-STR, *p* = 0.291). A-STR patients had smaller tenting height (TH) (10 ± 4 mm vs. 12 ± 7 mm, *p* = 0.023), larger end-diastolic tricuspid annulus area (9 ± 2 cm^2^ vs. 7 ± 6 cm^2^/m^2^, *p* = 0.007), smaller right ventricular (RV) end-diastolic volumes (72 ± 27 ml/m^2^ vs. 92 ± 38 ml/m^2^; *p* = 0.001), and better RV longitudinal function (18 ± 7 mm vs. 16 ± 6 mm; *p* = 0.126 for TAPSE, and −21 ± 5% vs. −18 ± 5%; *p* = 0.006, for RV free-wall longitudinal strain, RVFWLS) than V-STR patients. Conversely, RV ejection fraction (RVEF, 48 ± 10% vs. 46 ± 11%, *p* = 0.257) and maximal right atrial volumes (64 ± 38 ml/m^2^ vs. 55 ± 23 ml/m^2^, *p* = 0.327) were similar between the two groups. After a median follow-up of 10 months, patients with V-STR had a 2.7-fold higher risk (HR: 2.7, 95% CI 95% = 1.3–5.7) of experiencing the combined endpoint than A-STR patients. The factors related to outcomes resulted different between the two STR phenotypes: TR-severity (HR: 5.8, CI 95% = 1, 4–25, *P* = 0.019) in A-STR patients; TR severity (HR 2.9, 95% CI 1.4–6.3, *p* = 0.005), RVEF (HR: 0.97, 95% CI 0.94–0.99, *p* = 0.044), and RVFWLS (HR: 0.93, 95% CI 0.85–0.98, *p* = 0.009) in V-STR.

**Conclusion:**

Almost one-third of patients referred to the echocardiography laboratory for significant STR have A-STR. A-STR patients had a lower incidence of the combined endpoint than V-STR patients. Moreover, while TR severity was the only independent factor associated to outcome in A-STR patients, TR severity and RV function were independently associated with outcome in V-STR patients.

## Introduction

Secondary tricuspid regurgitation (STR) represents more than 90% of clinically relevant TR (1, 2) and is defined as TR that occurs in structurally normal tricuspid valve (TV) leaflets whose etiology is ascribed to tricuspid annular (TA) dilatation with or without leaflet tethering (3, 4).

Although the most common and well-known etiology of STR is the right ventricular (RV) dysfunction secondary to left-sided heart disease and pulmonary hypertension (PH), “isolated-TR” (TR not related to left-side heart disease or to significant pulmonary artery hypertension) has recently emerged as an important “phenotype” of STR. The prevalence of this phenotype of STR is growing because of the aging of the general population (1), and it develops mostly because of the right atrial (RA) dilatation associated with atrial fibrillation (AF) (5–8). The clarification of the pathogenic connection with AF and tricuspid annulus (TA) dilatation (8–10) has meant that this form of STR is currently defined as “*atrial”* STR (A-STR) (11).

Not surprisingly, the most recent guidelines about the management of heart valve diseases suggest that A-STR should be separated from the other forms of STR (12, 13) since the mechanisms underlying these two STR phenotypes have been hypothesized to be different (14). Notwithstanding, the recently published studies, including STR patients, do not distinguish between the A-STR and the “ventricular” STR (V-STR) phenotypes (15–19) and, to the best of our knowledge, only one echocardiographic study examined the differences in geometry of the right heart structures between patients with A-STR and V-STR (20). Furthermore, since A-STR has long been under-recognized as a distinct phenotype of STR, its prognosis and prognostic predictors remain to be clarified, especially in comparison with V-STR, in which the prognosis is also associated with the underlying RV function and/or associated left heart conditions (18, 19, 21). Accordingly, we tested the hypothesis that the prognosis and the prognostic correlates differ between patients with A-STR and V-STR.

## Materials and methods

### Study population and outcomes

Consecutive patients referred for echocardiography between 2016 and 2021 with the first diagnosis of moderate or severe STR were included in a prospective observational study (FUTURE 3DECHO). According to the most recent guidelines of the American Heart Association/American College of Cardiology (12), patients with moderate to severe STR were classified as A-STR when having AF, left ventricular ejection fraction > 60%, pulmonary artery systolic pressure (PASP) < 50 mm Hg, no left-sided valve disease, and normal-appearing tricuspid valve leaflets. Patients with STR not fitting all these three criteria were defined as having ventricular STR (V-STR) (12). Informed consent was collected from all subjects and the study was approved by the Istituto Auxologico Ethics Committee (record #2020_04_21_06, approved on April 21, 2020). The data collection and the echocardiographic analysis, as well as the follow-up, were performed by trained echocardiographers according to the best clinical practice and following the most recent recommendations (4, 22–24). Exclusion criteria were: poor echocardiographic image quality, a pacemaker or implantable cardioverter-defibrillator, organic tricuspid regurgitation, highly irregular cardiac rhythm (precluding the acquisition of multi-beat 3DE datasets with no stitching artifacts), pulmonary valve relevant pathologies or pulmonary artery stenosis; planned TV surgery or transcatheter intervention in the 3 months following the echocardiography evaluation.

### Echocardiography

**FIGURE 1:**
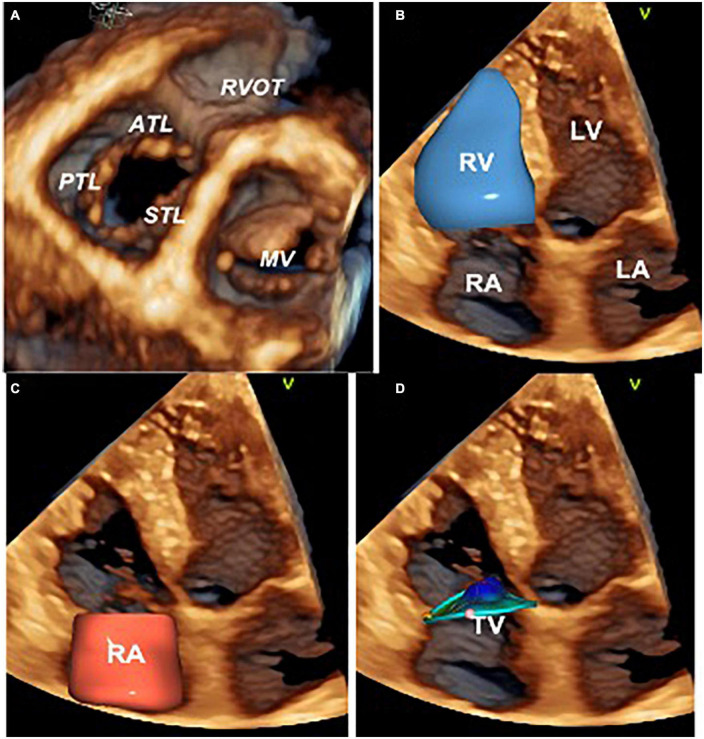
Three-dimensional echocardiography assessment of the tricuspid valve, right heart chambers, and tricuspid annulus. Ventricular view of the tricuspid and mitral valve leaflets by transthoracic three-dimensional echocardiography **(A)**. Surface rendering and volumetric analysis of the right ventricle **(B)** and right atrium **(C)**. Surface rendering and measurement of the tricuspid annulus size and shape with 4D AutoTVQ software package (GE Vingmed, Horten, NO) **(D)**. ATL, anterior tricuspid leaflet; LA, left atrium; LV, left ventricle; MV, mitral valve; RVOT, right ventricle outflow tract; PTL, posterior tricuspid leaflet; RV, right ventricle; RA, right atrium; STL, septal tricuspid leaflet; TV, tricuspid valve.

Study subjects underwent standard 2DE and Doppler studies using commercially available Vivid E9/E95 systems (GE Vingmed, Horten, Norway) equipped with M5S probe. In addition, multi-beat 3D datasets of the RA, TV, and RV were acquired from the apical approach using the 4V/4Vc probes. Images were digitally stored and analyzed offline using either EchoPAC 202 or 204 (GE Vingmed, Horten, Norway) by a single experienced researcher blinded to the medical history of the patients. Left ventricular (LV) volumes, LV ejection fraction (LVEF), PASP, and LV diastolic function were assessed in all the patients according to the most recent recommendations (4, 22–25). Conventional 2DE parameters of RA and RV size and function were obtained from the RV-focused apical view. 2DE TA diameter was measured from both the apical 4-chamber view and the RV-focused apical view at end-diastole (identified as the frame before the TV closure). RV free-wall longitudinal strain (RVFWLS) and 4-chamber RV strain (RV4CHLS) were obtained from the RV-focused apical view according to the current recommendations (26, 27). TR severity was graded as mild, moderate, or severe using the recommended multiparametric approach, which included: the average vena contracta (VC) width (measured in apical RV-focused and parasternal long-axis RV inflow views), the proximal isovelocity surface area (PISA) radius of the regurgitant jet at a Nyquist limit of 29 cm/s, the effective regurgitant orifice area (EROA) (23–25, 28, 29). To calculate the EROA and the regurgitant volume we used the PISA formula corrected for the TV leaflet tethering angle and the TR flow velocity (29). Full-volume 3DE acquisitions of the RV, TV, and RA were obtained from the RV-focused apical view using electrocardiogram gating over 4 to 6 consecutive cardiac cycles during a single breath-hold. Gain settings were optimized, and the sector width and the image depth were adjusted to maximize the temporal resolution. The RV end-diastolic (EDV) and end-systolic (ESV) volumes, and the RV ejection fraction (RVEF) were measured using 4D Auto RVQ (EchoPac 202 and 204, GE Vingmed, Horten, NO) (30). The absence of structural tricuspid valve (TV) diseases was checked by obtaining multiple cut-planes from the volume-rendered 3DE dataset of the valve. Finally, the size and the shape of the TA and leaflets’ coaptation position were evaluated using a dedicated software package (4D Auto TVQ, EchoPac v204, GE, Horten, Norway) (5, 26, 27; Figure 1).

### Follow-up and study endpoint

The primary endpoint of the study was the occurrence of death for any cause and/or hospitalization for heart failure. Information concerning survival and hospitalization were obtained at regular intervals through (*i*) telephone interviews with the patient, or if deceased, with family members; (*ii*) contact with the patient’s physician(s); and (*iii*) review of electronic medical records of regular outpatient visits and hospital admission records. Mortality status was verified independently through the Social Security Death Index and death certificates. Assignment of clinical events was performed by physicians unaware of the patients’ echocardiographic and clinical characteristics. For patients without events, the date of the last contact was used for survival analysis.

### Statistical analysis

The normal distribution of continuous variables was tested with Kolmogorov–Smirnov test. Continuous variables were reported as mean ± standard deviation (SD) and were compared using either the Student’s *t*-test or the Mann–Whitney test. Categorical variables were reported as counts and percentages and compared using the Fisher’s exact tests, as appropriate. Cox regression models were used to estimate the unadjusted and adjusted relative risk of clinical endpoints at follow-up. The results were shown as the hazard ratio (HR) with the corresponding 95% CI. Before running the multivariable analysis, we tested the correlation between echocardiographic parameters related to prognosis with the Pearson coefficient to exclude any multicollinearity. We included in the multivariable analysis the echocardiographic factors that resulted to be related to the survival at univariate analysis selected on the basis of their interaction and clinical relevance (i.e., *p* < 0.05 at univariate analysis). The cumulative incidence of all-cause mortality or HF hospitalization was estimated using the Kaplan–Meier method. All statistical analyses were performed using the SPSS software, version 20 (SPSS Inc., Chicago, IL, USA). A two-sided significance level of *p* < 0.05 was considered statistically significant.

## Results

### Clinical characteristics, two- and three-dimensional echocardiography parameters

**FIGURE 2:**
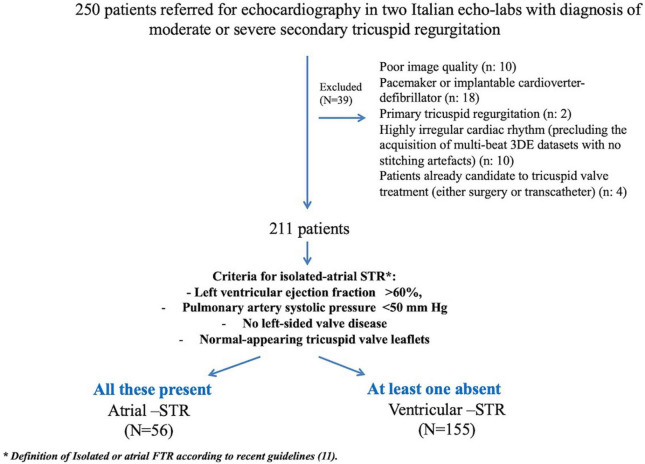
Study flow-chart. 3DE, three-dimensional echocardiography; A-STR, atrial secondary tricuspid regurgitation; RV, right ventricular; V-STR, ventricular secondary tricuspid regurgitation.

A total of 211 patients were included (Figure 2). Table 1 summarizes the clinal characteristics of our patients and the differences between patients with A-STR and V-STR. Compared with V-STR patients, A-STR patients (*n* = 26% of the study population) were significantly older and with a lower NYHA functional class. The prevalence of both ischemic heart disease and chronic obstructive pulmonary disease was higher in V-STR patients (Table 1).

**TABLE 1:** Clinical characteristics of patients with atrial and ventricular secondary tricuspid regurgitation.

Clinical factors		Overall the population (*n* = 211)	A-FTR (*n* = 56)	V-FTR (*n* = 155)	*P*-value
Age, years		74 ± 0.9	77 ± 9	73 ± 14	0.043
Women, n(%)		122 (57.8%)	34 (61%)	88 (72.1%)	0.609
Body surface area, m^2^		1.6 ± 0	1.6 ± 0.1	1.6 ± 0	0.653
Heart rate, bpm		77.1 ± 1.4	77.3 ± 2.5	77 ± 1.7	0.919
Systolic blood pressure, mmHg		129 ± 1.8	132.1 ± 2.7	128 ± 2.2	0.313
Diastolic blood pressure, mmHg		75.3 ± 1	76.6 ± 2	74.8 ± 1.1	0.443
Atrial fibrillation, n (%)		120 (57%)	56 (100%)	64 (41%)	0.198
NYHA functional class n(%)	I	95 (45%)	31 (55%)	64 (41%)	0.041
	II	80 (38%)	21 (38%)	59 (38%)	
	III	32 (15%)	4 (7%)	28 (18%)	
	IV	4 (2%)	0 (0%)	4 (3%)	
Diabetes, n (%)		36 (17%)	12 (22%)	23 (15%)	0.245
Arterial hypertension, n (%)		69 (33%)	18 (32%)	51 (33%)	0.898
Chronic obstructive pulmonary disease, n (%)		25 (12%)	3 (5%)	22 (14%)	0.086
Ischemic heart disease, n (%)		37 (17%)	5 (9%)	33 (21%)	0.031

A-STR, atrial secondary tricuspid regurgitation; NYHA, New York Heart Association; V-STR, ventricular secondary tricuspid regurgitation.

Table 2 summarizes the echocardiographic characteristics of our patients and the differences between patients with A-STR and V-STR. LVEF was higher in A-STR than in V-STR patients, and 7% of V-STR patients had LVEF < 40%. As expected, PASP was lower in A-STR patients (Table 2). The prevalence of severe STR was similar between A-STR and V-STR patients. However, V-STR patients showed larger vena contracta size (both the vena contracta width measured by 2DE and the vena contracta area measured by 3DE, Table 2). Moreover, TV remodeling parameters were significantly different between the two phenotypes. Patients with A-STR showed smaller tenting height and larger 3D end-diastolic tricuspid annulus area than V-STR patients (Table 2). RA volumes were similarly enlarged in A-STR and V-STR patients. The same was true for the RV basal end-diastolic diameter. Conversely, RV volumes were smaller in A-STR than in V-STR (Table 2). Global RV function (i.e., RVEF and TAPSE) were similar in A-STR and V-STR patients. Conversely, RV myocardial longitudinal deformation (reported as RVFWLS and RV4CLS) resulted significantly better in A-STR than in V-STR (Table 2). Similarly, RV-pulmonary artery coupling estimated by both TAPSE/PASP and RVFWLS/PASP was better in A-STR than in V-STR (Table 2).

**TABLE 2:** Echocardiographic characteristics of patients with atrial and ventricular secondary tricuspid regurgitation.

	Overall the population (*n* = 211)	A-STR (*n* = 56)	V-STR (*n* = 155)	*P*-value
**Echocardiographic parameters**				
**Left heart characteristics**				
LV ejection fraction,%	59 ± 7	65 ± 5	57 ± 9	< 0.001
LVejection fraction < 40%, n (%)	9 (5%)	0 (0%)	9 (7%)	0.031
Left atrial max volume, ml/m2	54 ± 19	51 ± 30	56 ± 23	0.258
**TR severity**				
Severe TR, n(%)	74 (35%)	16 (28%)	57 (37%)	0.291
2D-vena contracta average, mm	6.2 ± 3.4	5 ± 3	7 ± 4	0.022
3D-vena contracta area, cm^2^	1.1 ± 0.7	0.8 ± 0.5	1.2 ± 0.8	0.049
2D EROA (PISA), cm^2^	0.38 ± 0.29	0.33 ± 0.29	0.39 ± 0.25	0.221
Regurgitant volume, ml	33.7 ± 21.34	26 ± 19	37 ± 21	0.090
**Tricuspid valve remodeling**				
2D-tricuspid annulus diameter, mm/m^2^	24 ± 4	23 ± 4	24 ± 4	0.289
Tenting volume, ml	4.0 ± 1.8	3.9 ± 1.5	4.1 ± 1.9	0.665
Tenting height, mm	11 ± 6	10 ± 4	12 ± 7	0.023
End diastolic tricuspid annulus area, cm^2^	7 ± 5	9 ± 2	7 ± 6	0.007
**Right atrium**				
3D maximum volume index, ml/m2	60 ± 32	64 ± 38	55 ± 23	0.327
3D minimum volume index, ml/m2	49 ± 26	52 ± 25	47 ± 22	0.741
**Right ventricle**				
End-diastolic basal diameter, mm/m^2^	25.8 ± 6	24 ± 6	26 ± 6	0.071
End-diastolic volume, ml/m2	87 ± 36	72 ± 27	92 ± 38	0.001
End-systolic volume, ml/m2	49 ± 29	40 ± 20	52 ± 31	0.015
Stroke volume index, ml/m2	37 ± 15	34 ± 17	39 ± 14	0.041
Ejection fraction, %	47 ± 11	48 ± 10	46 ± 11	0.257
TAPSE, mm	17 ± 6	18 ± 7	16 ± 6	0.126
Free-wall longitudinal strain, %	19 ± 5	21 ± 5	18 ± 5	0.006
4-chamber strain, %	16 ± 5	17 ± 4	15 ± 5	0.035
Pulmonary artery systolic pressure PASP (mmHg)	48 ± 15	35 ± 18	53 ± 18	< 0.001
TAPSE/PASP. mm/mmHg	0.41 ± 0.23	0.57 ± 0.26	0.35 ± 0.19	< 0.001
RVFWLS/PASP%/mmHg	0.44 ± 0.22	0.64 ± 0.22	0.41 ± 0.19	< 0.001

2D, two-dimensional; 3D, three-dimensional; A-STR, atrial secondary tricuspid regurgitation; LV, left ventricle; TAPSE, tricuspid annular plane systolic excursion; V-STR, ventricular secondary tricuspid regurgitation.

### Clinical outcomes and associated echocardiography factors

**FIGURE 3:**
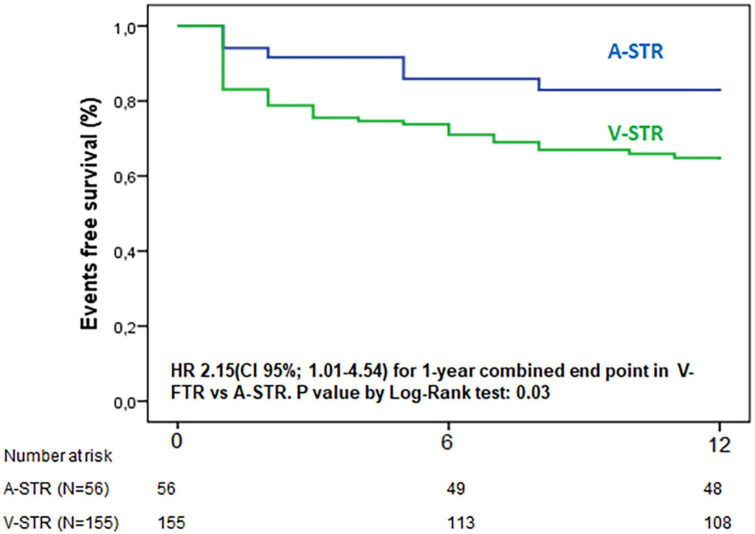
Kaplan-Meier curves for 1 year combined endpoint of all-cause death and hospitalization for heart failure. A-STR, atrial-secondary tricuspid regurgitation; HR, hazard Ratio; V-STR, ventricular secondary tricuspid regurgitation.

After a median follow-up of 10 months (IQR: 2–23), the rate of the composite endpoint was significantly different in the two groups (14% A-STR vs. 39% V-STR, *p* = 0.004), with a 2.15-fold significantly higher risk of 1 year combined endpoint for V-STR patients than A-STR (Figure 3).

Univariate Cox regression was performed for the whole study population, and then, separately, for each phenotype of STR to test the correlates of clinical outcomes (Table 3). All the indexes of TR severity were related to the composite endpoint in both groups. TH was associated with outcomes only in V-STR patients (*P* = 0.003). Conversely, the end-diastolic TA area was associated with outcomes only in A-STR (*P* = 0.025). Both RV end-diastolic and RA volumes were associated with outcomes at univariate analysis both in A-STR and V-STR patients. However, the RV function parameters were associated with outcomes only in V-STR patients (Table 3). Before running the multivariable regression analysis, we tested the correlation between echocardiographic variables. In A-STR patients, 3D maximal RA volume was related to the 3D end-diastolic TA area and the RV end-diastolic volume was related to the EROA. Accordingly, we included only TR severity and 3D maximal RA volume in our multivariable regression model, and TR severity resulted independently associated with the combined endpoint (HR: 5.8, CI 95%: 1.4–25, *P* = 0.019). In V-STR patients, a close correlation was found between 3D maximal RA volume and EROA, RV end-diastolic volume and TAPSE, and between RVEF, TAPSE, and TAPSE/PASP. Therefore, only TR severity, RVFWLS, RVEF, and 3D maximal RA volume were included in the final multivariable regression model (Table 4). TR severity (HR 2.9, CI 95% 1.4–6.3, *P* = 0.005 for TR severe), RVEF (HR: 0.97, CI 95%: 0.94–0.99, *P* = 0.044), and RVFWLS (HR: 0.93, CI 95%: 0.85–0.98, *P* = 0.009) resulted independently associated with outcomes.

**TABLE 3:** Univariate Cox regression analysis of factors related to the combined endpoint in the whole cohort and in the 2 distinct phenotypes of STR.

	Overall population (*n*: 210)		Ventricular-STR (*n*: 155)		Atrial-STR (*n*: 56)	
						
	HR (CI 95%)	*P*	HR (CI 95%)	*P*	HR (CI 95%)	*P*
**Clinical factors**						
Age, years	1.00 (0.98–1.02)	0.472	1.01 (0.99–1.03)	0.177	1.02 (0.96–1.09)	0.481
Body surface area, m^2^	0.99 (0.92–1.08)	0.386	1.00 (0.92–1.087)	0.971	0.94 (0.71–1.2)	0.937
Heart rate, bpm	1.01 (0.99–1.02)	0.123	1.03 (1.01–1.05)	0.031	1.01 (0.97–1.06)	0.778
Systolic blood pressure, mmHg	0.99 (0.98–1.02)	0.328	0.99 (0.98–1.01)	0.511	0.95 (0.91–1.02)	0.209
Atrial fibrillation, n(%)	1.7 (1.03–2.88)	0.037	2.38 (1.38–4.11)	0.002	–	–
NYHA functional class III-IV, n (%)	1.38 (0.78–2.45)	0.258	1.4 (0.80–2.62)	0.217	1.1 (0.56–1.99)	0.540
Diabetes, n (%)	0.73 (0.31–1.71)	0.291	0.67 (0.24–1.87)	0.441	1.4 (0.17–10)	0.818
Arterial hypertension, n (%)	0.64 (0.37–1.09)	0.252	0.88 (0.47–1.66)	0.709	0.46 (1.00–2.10)	0.309
Chronic obstructive pulmonary disease, n (%)	0.94 (0.43–2.05)	0.438	1.36 (0.58–3.19)	0.471	1.88 (0.24–14)	0.577
Ischemic heart disease, n (%)	0.58 (0.21–1.59)	0.625	1.08 (0.39–3.01)	0.870	1.48 (0.39–4.01)	0.899
Previous valve surgery, n (%)	0.88 (0.53–1.51)	0.524	0.53 (0.07–3.80)	0.524	–	–
**Echocardiographic parameters**						
**Left heart characteristics**						
LV ejection fraction, %	0.99 (0.96–1.01)	0.296	0.99 (0.96–1.02)	0.221	1.09 (0.95–1.28)	0.221
Reduced left ventricle ejection fraction less than 40% (n.%)	0.82 (0.20–3.37)	0.788	0.75 (0.18–3.08)	0.688	0.99 (0.96–1.02)	0.321
Left atrial max volume, ml/m2	0.99 (0.98–1.01)	0.921	1.00 (0.99–1.01)	0.998	0.99 (0.98–1.02)	0.852
Estimated elevated LV filling pressures	1.08 (0.49–2.34)	0.852	0.79 (0.33–1.95)	0.618	3.9 (0.76–7.81)	0.102
**TR parameters**						
TR grade severe versus moderate (n.%)	3.33 (2.07–5.35)	< 0.001	2.8 (1.60–4.80)	0.001	7.11 (2.39–21.11)	< 0.001
2D-vena contracta average. Mm	1.15 (1.09–1.20)	< 0.001	1.12 (1.07–1.19)	< 0.001	1.4 (1.2–1.7)	< 0.001
3D-vena contracta. cm^2^	1.52 (1.14–2.02)	< 0.001	1.37 (0.96–1.95)	0.08	8.2 (1.9–20)	< 0.001
2DEROA (PISA) angle corrected, cm^2^	3.00 (1.84–4.91)	< 0.001	2.40 (1.30–4.30)	0.003	2.00 (5.6–15.5)	0.001
Regurgitant volume angle-corrected, ml	1.03 (1.02–1.04)	< 0.001	1.02 (1.01–1.04)	< 0.001	1.06 (1.03–1.09)	< 0.001
Regurgitant fraction-angle corrected, n(%)	9.43 (4.42–20.01)	< 0.001	12 (4.90–30.00)	< 0.001	7.5 (1.8–20)	0.004
**Tricuspid valve remodeling**						
Tenting height, mm	1.09 (1.04–1.17)	0.002	1.11 (1.03–1.19)	0.003	1.03 (0.88–1.21)	0.691
Tenting volume, ml	1.12 (0.96–1.30)	0.139	1.12 (0.96–1.31)	0.154	1.11 (0.67–1.83)	0.681
Tricuspid annulus diameter, mm/m2	1.03 (0.99–1.06)	0.091	1.03 (0.99–1.07)	0.101	1.03 (0.96–1.11)	0.343
End diastolic tricuspid annulus area, cm^2^/m^2^	1.05 (0.97–1.12)	0.211	1.13 (1.02–1.27)	0.791	1.14 (1.02–1.27)	0.025
Mid systolic tricuspid annulus-area in, cm^2^/m^2^	1.00 (0.97–1.02)	0.967	1.00 (0.98–1.012)	0.982	1.01 (0.96–1.95)	0.836
**RA dimensions**						
3D-right atrial maximum volume, ml/m2	1.01 (1.00–1.02)	0.005	1.01 (1.00–1.02)	0.034	1.01 (1.00–1.02)	0.016
3D-right atrial minimum volume, ml/m2	1.02 (1.01–1.03)	< 0.001	1.02 (1.01–1.04)	0.001	1.03 (1.01–1.05)	0.016
**RV-dimension and function**						
End-diastolic basal diameter, mm/m^2^	1.08 (1.04–1.12)	< 0.001	1.07 (1.02–1.11)	0.004	1.15 (1.02–1.29)	0.018
End-diastolic volume, ml/m2	1.01 (1.00–1.01)	0.001	1.01 (1.00–1.01)	0.002	1.02 (1.00–1.04)	0.046
End-systolic volume, ml/m2	1.01 (1.00–1.02)	< 0.001	1.01 (1.00–1.02)	0.001	1.016 (0.99–1.04)	0.208
Stroke volume, ml/m2	1.02 (1.00–1.04)	0.016	1.01 (0.99–1.03)	0.227	1.07 (1.02–1.14)	0.013
PASP, mmHg	1.02 (1.01–1.03)	< 0.001	1.02 (1.01–1.03)	< 0.001	1.02 (0.99–1.04)	0.225
TAPSE, mm	0.93 (0.88–0.98)	0.004	0.92 (0.86–0.98)	0.008	0.97 (0.87–1.08)	0.598
Free-wall longitudinal strain, %	0.92 (0.88–0.96)	< 0.001	0.91 (0.87–0.96)	0.001	0.97 (0.87–1.07)	0.527
4-chamber strain, %	0.94 (0.89–0.98)	0.017	0.92 (0.87–0.98)	0.011	1.00 (0.89–1.13)	0.996
Ejection fraction, %	0.97 (0.95–0.99)	0.012	0.96 (0.94–0.99)	0.007	1.01 (0.95–1.07)	0.782
**Right ventriculo-arterial coupling surrogates**						
TAPSE/PASP, mm/mmHg	0.05 (0.01–0.22)	< 0.001	0.02 (0.01–0.16)	< 0.001	0.09 (0.01–1.25)	0.073
RVFWLS/SPAP%/mmHg	0.05 (0.01–0.19)	< 0.001	0.01 (0.01–0.07)	< 0.001	0.28 (0.02–3.09)	0.296

2D, two-dimensional; 3D, three-dimensional; A-STR, atrial secondary tricuspid regurgitation; PASP, pulmonary artery systolic pressure; RA, right atrium; RV, right ventricle; RVFWLS, Right ventricle free wall longitudinal strain; RVGLS, right ventricle global longitudinal strain; TA, tricuspid annulus; TR, tricuspid regurgitation; TAPSE, tricuspid annular plane systolic excursion; V-STR, ventricular secondary tricuspid regurgitation.

**TABLE 4:** Multivariable Cox regression to identify the variables associated with the combined endpoint in patients with atrial and ventricular secondary tricuspid regurgitation.

		HR (CI 95%)	*P*–value
V-STR patients	TR grade (severe versus moderate)	2.9 (1.4–6.3)	0.005
	Right ventricular free wall longitudinal strain (RVFWLS, –%)	0.93 (0.85–0.98)	0.009
	RV ejection fraction (%)	0.97 (0.94–0.99)	0.044
	3D max RAV index (ml/m2)	1.01 (0.99–1.02)	0.366
A-STR patients	3D-right atrial maximum volume (ml/m2)	1.01 (0.99–1.07)	0.34
	TR grade severe versus moderate	5.8 (1.4–25)	0.02

3D, three-dimensional; A-STR, atrial secondary tricuspid regurgitation; RA, right atrium; RV, right ventricle; RVFWLS, right ventricle free wall longitudinal strain; RVGLS, right ventricle global longitudinal strain; SPAP, Systolic pulmonary arterial pressure; TA, tricuspid annulus; TR, tricuspid regurgitation; TAPSE, tricuspid annular plane systolic excursion; V-STR, ventricular secondary tricuspid regurgitation.

## Discussion

The present is the first study aiming to compare the outcome and its associated factors among patients with moderate or severe STR, classified according to recent guidelines in A-STR and V-STR. Our results show that (*i*) patients with A-STR are older and less symptomatic than V-STR patients, with fewer leaflet tethering, larger TA size, and more preserved RV function and RV-PA coupling. (*ii*) A-STR patients have a significantly lower incidence of death and hospitalization for HF at follow-up. (*iii*) While STR severity is the only parameter independently associated with prognosis in A-STR, RV function (in addition to STR severity) had prognostic relevance in V-STR.

### Difference in outcomes and right chamber remodeling of atrial secondary tricuspid regurgitation and ventricular secondary tricuspid regurgitation

In the last years, the growing interest in interventional treatment of TR has raised the need of having a precise characterization of patients with significant STR, and a nomenclature that could classify patients with STR according to the main pathogenetic mechanism. Only recently, the clinical and echocardiographic parameters to be used to identify A-STR have been reported (12, 13, 31). Accordingly, A-STR has been recognized as a distinct pathophysiological entity, and its mechanisms have begun to be studied (5–7, 32, 33). Also, A-STR is not a benign condition. Patients with isolated or A-STR are frequently hospitalized for HF and experience excess mortality. Elevated right atrial pressure and renal dysfunction are associated with mortality. This poor outcome may have implications for timing of intervention (34). Abe et al. reported that, in AF patients with preserved LVEF, the combination of mitral and STR was associated with a combined endpoint that include: cardiac death, admission due to heart failure, or mitral and/or tricuspid valve surgery (35). Both the mitral and the TR grade were independently related to the combined endpoint, irrespectively from the other echocardiographic parameters (35).

Another study confirmed the independent role of STR in long-standing AF patients for predicting HF hospitalization and death for any cause (36). Finally, a recent study based on a clustering analysis (and no *a priori* assumption) identified three distinct phenotypes (clusters) of STR based on RV and RA volumes and function: cluster 1 included patients with better right ventricular, left ventricular, and right atrial function; cluster 2 with reduced RV and RA strain despite similar sizes; cluster 3 patients with severely dilated heart chambers associated to RV and RA dysfunctions. These 3 phenotypes were associated with different outcomes and, under non-interventional management, the phenotype corresponding to preserved RV size and preserved RA and RV functions had less incidence of the combined endpoint (death and HF hospitalization) (17).

In our study, A-STR patients were identified using the criteria proposed in the last ACC/AHA guidelines (12). Using this definition, A-STR patients were found to be older than patients with V-STR but with less advanced functional impairment. Moreover, A-STR patients had a 2.7 lower risk of death for any cause and HF hospitalization compared to V-STR patients. Regarding the different echocardiographic remodeling observed in the 2 population, interesting results regarding RA, TA, and RV remodeling were found, in line with previous evidence (8, 20, 37).

Recently, a study from our group sought to analyze the TV geometry in a cohort of patients with any degree of TR compared with a control cohort and classified in A- and V-STR on the base of an echocardiographic definition (20). Contrary to our results, in that study, A-STR patients showed larger RAV min compared to V-STR. The fact that in our study the RA dilatation is similar between the 2 groups may depend on different definitions of A- and V-STR in the methods, being the RA dimension not a criterium in our study; secondly, our study included only patients with at least moderate STR (more severe STR). Therefore, the results of these 2 studies should be considered as a continuum in the spectrum of different grades of severity of STR, reaffirming the predominant role of RA and TA dilatation in determining the grade of TR in A-STR (20). The 3D-assessed TA area (both at end diastole and mid-systole) was larger in A-STR than in V-STR and, therefore, despite lower tenting height, the tenting volume was similar to V-STR patients. The assessment of TA size using 2DE linear dimensions was unable to detect this important difference about the remodeling of the TA between these two phenotypes of STR, in line with previous evidence (38). At the same way, also the RV-basal linear end-diastolic diameter was similar in the 2 groups of A-STR and V-STR but lower RV volumes were found in the A-STR. All these results are an important confirmation of the fact that RA, TA and RV size assessment by two-dimensional echocardiography (2DE) and linear methods, has important limitations in patients with STR and should be integrated with 3DE. Finally, regarding RV functional assessment, RVEF was not significantly different in the 2 groups; conversely RVFWLS was higher in patients with A-STR. These results reflect the stronger dependency of RVEF from volume overload of significant STR, as already demonstrated (16, 39, 40).

### Echocardiographic factors related to outcomes in atrial secondary tricuspid regurgitation and ventricular secondary tricuspid regurgitation

The novelty of the present study was to identify the parameters associated with outcomes in A-STR and V-STR patients. In the A-STR cohort, the prognosis was independently associated only with the severity of STR rather than the size and the function of the RV and RA. The fact that none of the parameters of RV function was associated with the incidence of the combined endpoint agrees with the results of a previous study on patients with isolated TR (without mention regarding the AF link), in which RV dysfunction was not related to clinical outcomes, but the RA pressure and the venous congestion did (34). As a clinical perspective, the independent role of the severity of TR confirms that in patients with A-STR the reduction of TR, *per se*, should represent the main goal of the treatment.

Conversely, in patients with V-STR, RV function, in addition to STR severity, was associated with outcome. Recently, in a large population analysis, the importance of RV function and PASP on the prognosis of patients with STR has been explored (19). In this study, the higher the extent of the RV damage and the pulmonary pressure, the lower the relative impact of significant STR. In our study, STR severity and RV functional parameters were found to be independently associated with prognosis in the cohort of V-STR patients. These results further reflect the intrinsic pathogenetic relationship between STR and RV functional impairment, which are self-maintaining as if in a vicious cycle, and, again, highlight the importance of reducing STR grade to achieve disruption of this vicious cycle, even in patients with RV dysfunction (41–44).

The results of the present study should be considered in the “spectrum” of the recent evidence from the studies comparing the atrial and ventricular etiologies of secondary mitral regurgitation (SMR) (45, 46). Indeed, these data confirm that the two phenotypes of atrioventricular valve regurgitation are characterized by different clinical and echocardiographic characteristics, as well as different prognosis. As speculated in the setting of secondary mitral regurgitation, our results may have important implications for patients’ selection for transcatheter treatment of STR, underlying the relative importance and the heterogeneity of different remodeling parameters in prognostic stratification.

### Study limitations

The limitations of the present study are inherent to its observational design, which allows only hypothesis making and no causal inference. In addition, the sample size was relatively small. However, we selected patients with complete 3D and good quality datasets to provide robust parameters to characterize A-ATR and V-STR patients fully. The relatively small size of the study population and the low rate of events are limitations that should be accounted for in our survival analysis. Medical records about medical treatment were somehow imprecise and incomplete and were not included in the present analysis and evaluated. Furthermore, although our findings align with current literature, we cannot exclude a selection bias affecting our results. Studies with a larger cohort of patients and a longer follow-up may therefore be needed to confirm our results.

## Data availability statement

The original contributions presented in this study are included in the article/supplementary material, further inquiries can be directed to the corresponding author.

## Ethics statement

The studies involving human participants were reviewed and approved by the study was approved by the Istituto Auxologico. The patients/participants provided their written informed consent to participate in this study.

## Author contributions

MG and FH contributed to conception and design of the study, organized the database, performed the statistical analysis, and wrote the first draft of the manuscript. LB and DM reviewed the results and the manuscript and added his special contribution as expert of field. All authors contributed to manuscript revision, read, and approved the submitted version.
