# Corrigendum: The atrial secondary tricuspid regurgitation is associated to more favorable outcome than the ventricular phenotype

**DOI:** 10.3389/fcvm.2023.1169907

**Published:** 2023-03-13

**Authors:** Mara Gavazzoni, Francesca Heilbron, Luigi P. Badano, Noela Radu, Andrea Cascella, Michele Tomaselli, Francesco Perelli, Sergio Caravita, Claudia Baratto, Gianfranco Parati, Denisa Muraru

**Affiliations:** ^1^ Department of Cardiology, Istituto Auxologico Italiano, IRCCS, Milan, Italy; ^2^ Department of Medicine and Surgery, University of Milano-Bicocca, Milan, Italy; ^3^ University of Medicine and Pharmacy Carol Davila Bucharest, Emergency University Hospital Bucharest, Bucharest, Romania; ^4^ Department of Management, Information and Production Engineering, University of Bergamo, Dalmine, Italy

**Keywords:** atrial secondary tricuspid regurgitation, atrial fibrillation, heart failure hospitalizations, secondary tricuspid regurgitation, prognosis

A Corrigendum on The atrial secondary tricuspid regurgitation is associated to more favorable outcome than the ventricular phenotype By Gavazzoni M, Heilbron F, Badano LP, Radu N, Cascella A, Tomaselli M, Perelli F, Caravita S, Baratto C, Parati G, Muraru D. (2022) Front. Cardiovasc. Med. 9:1022755. doi: 10.3389/fcvm.2022.1022755

In the published article, there was an error in affiliation (2) for the following 3 authors: Mara Gavazzoni, Michele Tomaselli, Claudia Baratto. The affiliation (2) “Department of Medicine and Surgery, University of Milano-Bicocca, Milan, Italy” does not apply to these three authors. The only affiliation for these authors should be (1): “Department of Cardiology, Istituto Auxologico Italiano, IRCCS, Milan, Italy”, as displayed in the new version of the paper.

The authors apologize for this error and state that this does not change the scientific conclusions of the article in any way. The original article has been updated.

